# OA11.02. A model of integrative care for low back pain

**DOI:** 10.1186/1472-6882-12-S1-O42

**Published:** 2012-06-12

**Authors:** D Eisenberg, J Buring, A Hrbek, R Davis, M Connelly, D Cherkin, D Levy, M Cunningham, B O'Connor, D Post

**Affiliations:** 1 Harvard Medical School, Chestnut Hill, USA; 2 Brigham and Women's Hospital, Boston, USA; 3 Beth Israel Deaconess Medical Center, Boston, USA; 4 Group Health Research Institute, Seattle, USA; 5 Brown University, Providence, USA

## Purpose

While previous studies focused on the effectiveness of individual complementary and alternative medical (CAM) therapies, the value of providing patients access to an integrated program involving multiple CAM and conventional therapies remains unknown. Our objective is to explore the feasibility and effects of a model of multidisciplinary integrative care for subacute low back pain (LBP) in an academic teaching hospital.

## Methods

Study design was a pilot randomized trial comparing an individualized program of integrative care (IC) plus usual care to usual care (UC) alone for adults with LBP. Twenty individuals with LBP of 3-12 weeks duration were recruited from an occupational health clinic and community health center. Participants were randomized to 12 weeks of individualized IC plus usual care vs UC alone. Integrative care was provided by a trained multidisciplinary team offering CAM therapies and conventional medical care. Outcome measures were symptoms (pain, bothersomeness), functional status (Roland-Morris score), SF-12, worry, and difficulty performing 3 self-selected activities.

## Results

Over 12 weeks, participants in the IC group had a median of 12.0 visits (range 5-25). IC participants experienced significantly greater improvements at 12 weeks than those receiving UC alone in symptom bothersomeness (p=0.02) and pain (p=0.005), and showed greater improvement in functional status (p=0.08). Rates of improvement were greater for patients in IC than UC in functional status (p=0.02), bothersomeness (p=0.002), and pain scores (p=0.001). Secondary outcomes of self-selected most challenging activity, worry and the SF-12 also showed improvement in the IC group at 12 weeks. These differences persisted at 26 weeks, but were no longer statistically significant.

## Conclusion

It was feasible for a multidisciplinary, outpatient integrative care team to deliver coordinated, individualized intervention to patients with subacute LBP. Results showed a promising trend for benefit of treating patients with persistent LBP with this integrative care model, and warrant evaluation in a full-scale study.

